# Effectiveness of different intrusion modes of maxillary anterior teeth with mini-implants in clear aligner treatment: a three-dimensional finite element analysis

**DOI:** 10.1186/s12903-024-04537-7

**Published:** 2024-07-02

**Authors:** Tian Xiao, Jing-yuan Su, Jie Lei, Xin Zhang, Jian Yu, Xiu-ping Nie, Qiao-hui Ying, Jun-xiang Hou, Jie Guo

**Affiliations:** 1 Department of Orthodontics, School and Hospital of Stomatology, Cheeloo College of Medicine, Shandong Key Laboratory of Oral Tissue Regeneration & Shandong Engineering Research Center of Dental Materials and Oral Tissue Regeneration & Shandong Provincial Clinical Research Center for Oral Diseases, Shandong University, No.44-1 Wenhua Road West, Jinan, Shandong 250012 China; 2 Department of Radiology, School and Hospital of Stomatology, Cheeloo College of Medicine, Shandong Key Laboratory of Oral Tissue Regeneration & Shandong Engineering Research Center of Dental Materials and Oral Tissue Regeneration & Shandong Provincial Clinical Research Center for Oral Diseases, Shandong University, No.44-1 Wenhua Road West, Jinan, Shandong 250012 China; 3 R&D center, Wuxi EA Medical Instruments Technologies Ltd, Wuxi, 214174 China

**Keywords:** Intrusion modes, Mini-implant, Clear aligner, Finite element analysis

## Abstract

**Background:**

The intrusion of maxillary anterior teeth is often required and there are various intrusion modes with mini-implants in clear aligner treatment. The objective of this study was to evaluate the effectiveness of maxillary anterior teeth intrusion with different intrusion modes, aiming to provide references for precise and safe intrusion movements in clinical practice.

**Methods:**

Cone-beam computed tomography and intraoral optical scanning data of a patient were collected. Finite element models of the maxilla, maxillary dentition, periodontal ligaments (PDLs), clear aligner (CA), attachments, and mini-implants were established. Different intrusion modes of the maxillary anterior teeth were simulated by changing the mini-implant site (between central incisors, between central and lateral incisor, between lateral incisor and canine), loading site (between central incisors, on central incisor, between central and lateral incisor, between lateral incisor and canine), and loading mode (labial loading and labiolingual loading). Ten conditions were generated and intrusive forces of 100 g were applied totally. Then displacement tendency of the maxillary anterior teeth and CA, and stress of the PDLs were analyzed.

**Results:**

For the central incisor under condition L14 and for the canine under conditions L11, L13, L23, and L33, the intrusion amount was negative. Under other conditions, the intrusion amount was positive. The labiolingual angulation of maxillary anterior teeth exhibited positive changes under all conditions, with greater changes under linguoincisal loading. The mesiodistal angulation of canine exhibited positive changes under labial loading, while negative changes under linguoincisal loading except for condition L14.

**Conclusions:**

The intrusion amount, labiolingual and mesiodistal angulations of the maxillary anterior teeth were affected by the mini-implant site, loading site, and loading mode. Labial and linguoincisal loading may have opposite effects on the intrusion amount of maxillary anterior teeth and the mesiodistal angulation of canine. The labiolingual angulation of the maxillary incisors would increase under all intrusion modes, with greater increases under linguoincisal loading.

## Background

There are numerous clinical situations requiring the intrusion of maxillary anterior teeth, such as gummy smile and deep overbite. The gummy smile not only affects facial aesthetics, but also induces negative emotions during social interaction [1, 2]. For those resulting from anterior dentoalveolar extrusion and vertical maxillary excess, it can be improved by the intrusion of maxillary anterior teeth [1, 2]. Additionally, the deep overbite is a common malocclusion, which can affect facial aesthetics, occlusal function, and overall oral health [3]. In cases of skeletal Class II and vertical growth pattern with deep overbite, the intrusion of maxillary anterior teeth not only facilitates bite opening but also creates space for counterclockwise rotation of the mandible, which is crucial for the improvement of facial profile [1, 4].

Traditional methods for intruding maxillary anterior teeth, including J-hook headgear, continuous arch, segmental intrusion arches, Rickett’s utility arch, etc., have various limitations. J-hook headgear causes strong discomfort and requires patient compliance [4]. Other methods are less effective and may lead to the extrusion of posterior teeth [3]. Researchers have confirmed that the application of mini-implants can effectively facilitate the intrusion of maxillary anterior teeth and achieve true intrusion [3]. Mini-implants have the advantages of immediate force application, clear biomechanics, and diversity of implantation site and force direction [3, 5]. However, different mini-implant sites and loading sites will lead to different force lines and then different effects. In fixed orthodontic treatment, some studies have reported that mini-implants can be placed between maxillary central incisors [6], central incisor and lateral incisor [1], lateral incisor and canine [6, 7]. Mini-implants between central incisors can effectively increase the labiolingual angulation of maxillary anterior teeth, while mini-implants between lateral incisor and canine are advantageous for achieving true intrusion [6].

Recently, clear aligner (CA) has become an increasingly important part of orthodontic treatment due to its advantages of aesthetics, comfort, and less impact on social life [8]. However, CA is not effective in intruding the anterior teeth. Some researchers proposed that the intrusion of anterior teeth is one of the most difficult movements to achieve with CA [9], with studies showing an average efficiency of 51.1% and an average true intrusion amount of 0.9 mm [10]. It has been shown that combining CA with mini-implants can effectively improve the intrusion efficiency of maxillary anterior teeth [11, 12]. Nevertheless, the biomechanics involved is more complicated due to the different force application patterns and material characteristics [13]. The different effects caused by different intrusion modes in clear aligner treatment remain unclear and lack reliable references in clinical practice.

In this study, finite element analysis (FEA) was used to investigate the effectiveness of different intrusion modes of maxillary anterior teeth with mini-implants, in order to provide references for precise and safe intrusion movements in clear aligner treatment. And the following null hypotheses were proposed: (1) Different intrusion modes do not affect the displacement tendency of the maxillary anterior teeth and CA. (2) Different intrusion modes do not affect the stress of the PDLs.

## Methods

A 22-year-old female patient was selected as the research subject. The patient met the following inclusion criteria: (1) permanent dentition with basically symmetrical dental arch; (2) normal labiolingual angulation of maxillary anterior teeth (U1-SN = 100°); (3) normal morphology and crown-root ratio of maxillary anterior teeth; (4) no apparent crowding; (5) no periodontal disease and orthodontic history; (6) consonant smile arc.

Cone-beam computed tomography (CBCT) and intraoral optical scanning data were collected for the reconstruction of digital models. Firstly, CBCT data were imported into Mimics (version 21.0; Materialise, Leuven, Belgium), where three-dimensional models of the maxilla and maxillary dentition were established based on the different gray-scale values of different tissues. Next, the models were imported into Geomagic Studio (version 2016; 3D systems, USA) for smoothing and noise reduction. Then, the iterative closest point technique was used to precisely align the reconstructed model with the intraoral scan model, resulting in a high-precision dentition model.

According to the clinical situation, vertical rectangular attachments (2 × 4 × 1 mm) were designed on the bilateral maxillary canines, and horizontal rectangular attachments (3 × 2 × 1 mm) were designed on the premolars. The periodontal ligaments (PDLs) were modeled on root shape with uniform thickness of 0.25 mm [8]. Based on Masterforce (Angelalign Technology Inc., Shanghai, China), a more realistic CA model with nonuniform thickness was established by simulating the thermoforming process in Abaqus (version 2016; Simula, USA) [14]. Then, all components were assembled to generate the final model. All components were meshed as tetrahedral elements, with the mesh size of 0.1 mm for attachments and 0.3 mm for other components. The number of nodes and elements of each component was listed in Table 1. The PDL models were set as linear elastic materials to achieve an optimal balance between accuracy and computational efficiency. Other components were set to be isotropic, homogeneous, and linear elastic. The material properties, as listed in Table 2, were determined according to previous studies [8, 15–17].

**Table 1:** Number of nodes and elements of the components

Component	Elements	Nodes
Dentition	252,572	126,328
PDL	78,042	79,300
Cancellous bone	353,090	79,966
Cortical bone	617,770	151,190
Clear aligner	79,626	79,626
Mini-implant	25,856	12,932

**Table 2:** Material properties

Component	Young‘s modulus (MPa)	Poisson‘s ratio
Teeth	19,600	0.30
PDL	0.69	0.45
Cancellous bone	1500	0.30
Cortical bone	14,700	0.30
Clear aligner	528	0.36
Attachment	12,500	0.36
Mini-implant	103,000	0.33

The base of the maxilla was fixed, that is, zero displacement and zero rotation were set. By sharing finite element nodes, bonded contacts were set between the PDLs and bone, the PDLs and teeth, the mini-implants and bone, as well as the attachments and teeth. Surface-to-surface contacts were set between the CA and teeth, as well as the CA and attachments, with the frictional coefficient of 0.2 [15].

**Fig. 1:**
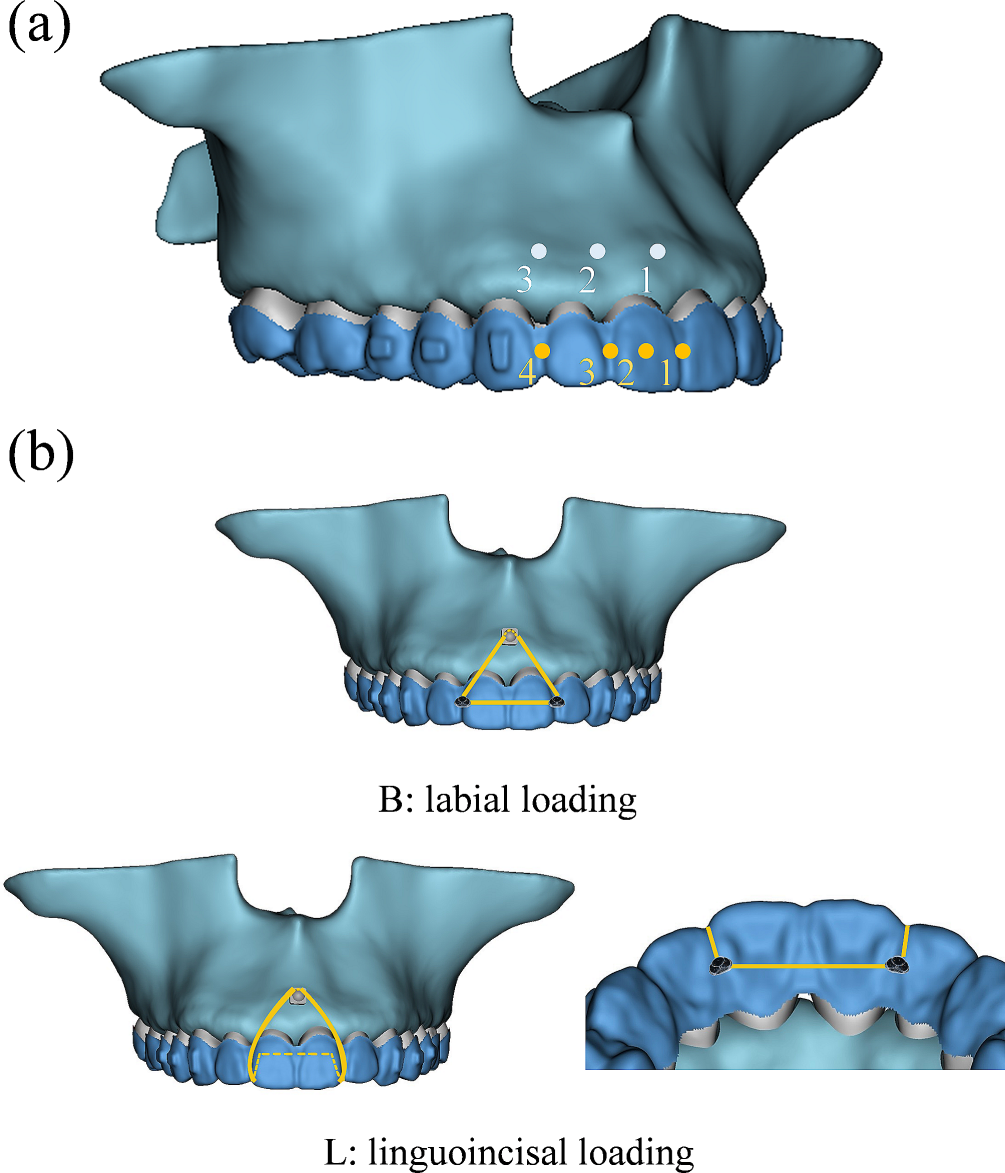
Different mini-implant sites, loading sites and loading modes. (**a**) Different mini-implant sites and loading sites. (**b**) Different loading modes

**Fig. 2:**
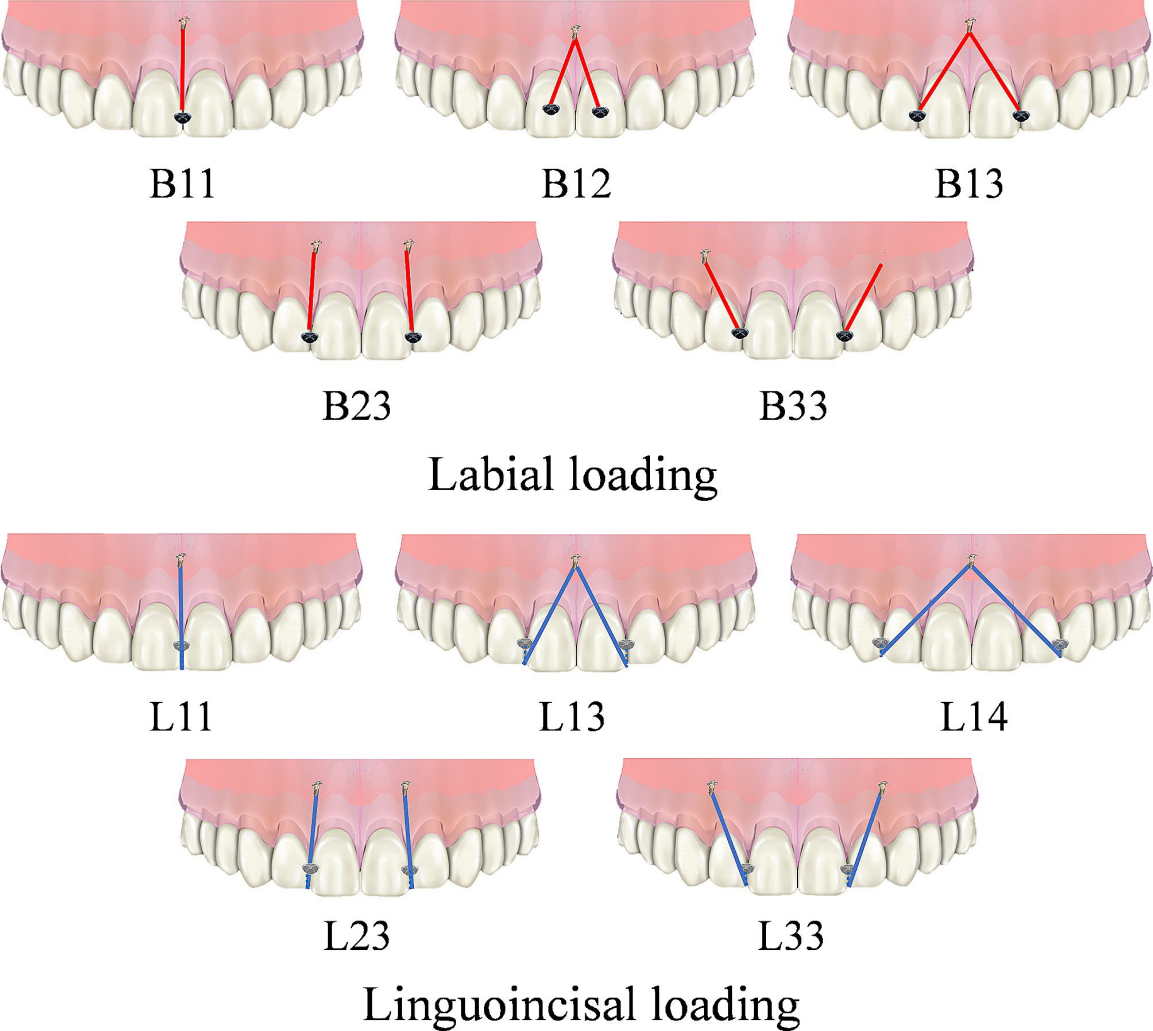
Different conditions. Each condition was named in the form of “letter + number + number”, where the letter represents the loading mode, the first number represents the mini-implant site, and the second number represents the loading site. The red lines represent the elastic bands under labial loading, and the blue lines represent the elastic bands under linguoincisal loading

As displayed in Fig. 1, three kinds of mini-implant site were designed: between central incisors, between central and lateral incisor, and between lateral incisor and canine, which were marked as “1, 2, 3” from mesial to distal respectively. Four kinds of loading site were designed: between central incisors, on central incisor, between central and lateral incisor, and between lateral incisor and canine, which were marked as “1, 2, 3, 4” from mesial to distal respectively. Two kinds of loading mode were designed, with labial loading marked as “B” and linguoincisal loading marked as “L”. For labial loading, elastic bands were worn from the mini-implants to the labial side of CA. For linguoincisal loading, elastic bands were worn from the mini-implants to the lingual side of CA, passing beneath the incisal edges. As displayed in Fig. 2, a total of ten conditions were generated. Under each condition, one or two loading sites were designed, and the corresponding force value of each site was set 100 g and 50 g respectively to ensure the total force was 100 g [17, 18].

The center of resistance (CR) points of maxillary anterior teeth were defined as the position of 1/3 root length measured apically from the alveolar crest [16]. The maxillary occlusal plane was defined as the plane passing through the midpoint of the central incisors and the mesial buccal cusps of the first molars [9]. The tooth axis was defined as the line connecting the incisal center or canine cusp and the CR point [19].

**Fig. 3:**
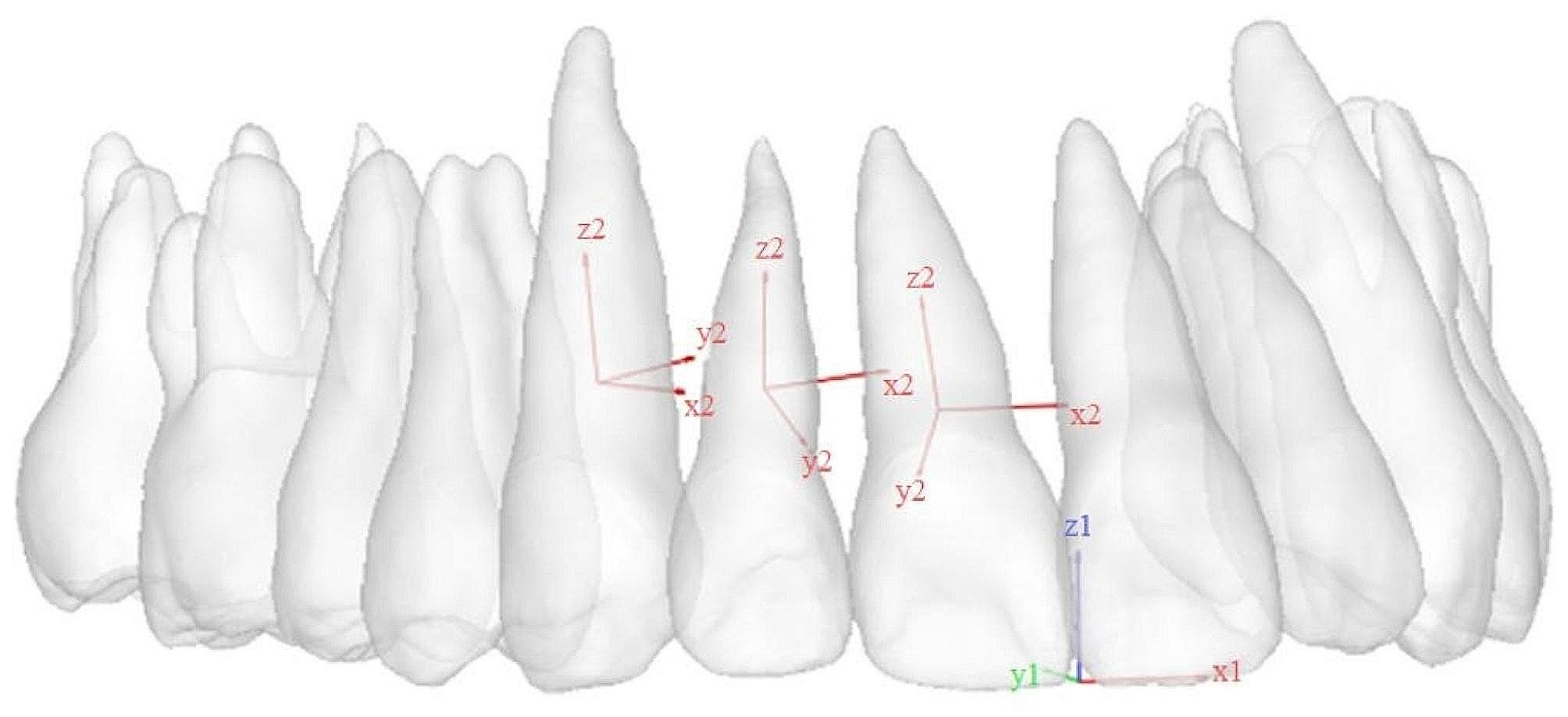
The 3D coordinate systems. x1y1z1: the global coordinate; x2y2z2: the local coordinate

Since the left and right sides of the model were basically symmetrical, only the right side was analyzed. As displayed in Fig. 3, two types of coordinates were designed:

1. the global coordinate (x1y1z1) was established relative to the entire model, with the origin set at the midpoint of the central incisors. The z1-axis represented the vertical direction, with the positive direction pointing apically perpendicular to the occlusal plane.
2. the local coordinates (x2y2z2) were established individually for each anterior tooth, with the origins set at the CR points of each tooth. The x2-axis represented the direction of the line connecting mesiodistal contact points; the y2-axis represented the labiolingual direction; and the z2-axis represented the direction of tooth axis.

The measuring items included:

1. the intrusion amount of maxillary anterior teeth: it was defined as the displacement of CR points in z1-direction, with positive values for intrusion;
2. the change in labiolingual angulation of maxillary anterior teeth: it was defined as the angle of tooth axes on the sagittal plane under the local coordinates before and after loading, with positive values for crown labial tipping;
3. the change in mesiodistal angulation of canine: it was defined as the angle of tooth axes on the coronal plane under the local coordinates before and after loading, with positive values for crown mesial tipping;
4. the initial displacement tendency of CA;
5. the maximum principal stress of PDLs: negative values represented compressive stress and positive values represented tensile stress.

## Results

### Effects of different intrusion modes on the intrusion amount

**Fig. 4:**
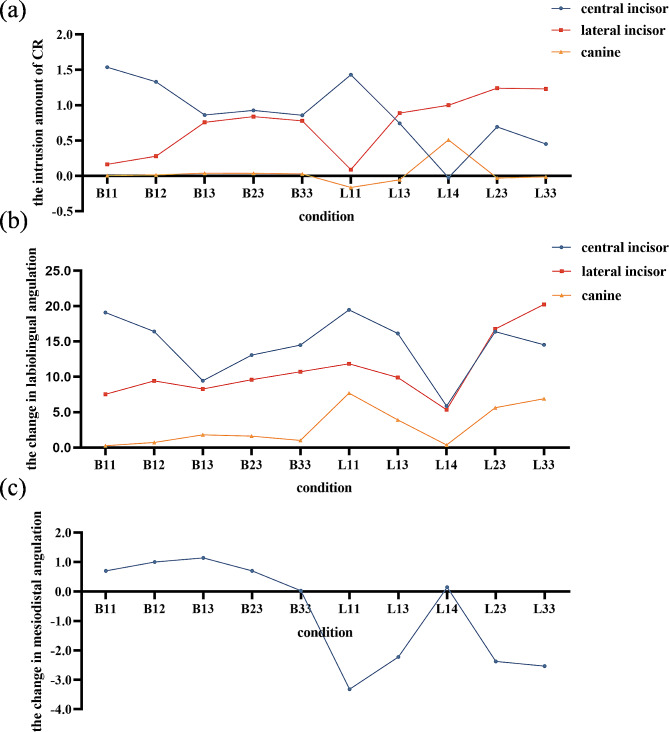
Displacement tendencies of maxillary anterior teeth. (**a**) Intrusion amount of maxillary anterior teeth (µm). (**b**) Changes in labiolingual angulation of maxillary anterior teeth (second). (**c**) Changes in mesiodistal angulation of canine (second)

As displayed in Fig. 4 (a), the intrusion amount of the lateral incisor was positive under all conditions. The intrusion amount of the central incisor and canine was positive under labial loading, but the intrusion amount of the central incisor was negative under L14 (-0.024 μm). The intrusion amount of the canine was negative under L11, L13, L23, and L33, with the minimum observed under L11 (-0.163 μm). The intrusion amount of the central incisor was the highest under B11 (1.535 μm), followed by L11 (1.430 μm), B12 (1.330 μm), B23 (0.927 μm), and B13 (0.861 μm); The intrusion amount of the lateral incisor was the highest under L23 (1.240 μm), followed by L33 (1.230 μm), L14 (0.998 μm), L13 (0.888 μm), and B23 (0.838 μm); The intrusion amount of the canine was the highest under L14 (0.509 μm), followed by B13 (0.037 μm), B23 (0.036 μm), B33 (0.027 μm), and B12 (0.013 μm).

### Effects of different intrusion modes on the labiolingual angulation

As displayed in Fig. 4 (b), the changes in labiolingual angulation were positive under all conditions. For the central incisor, the maximum was observed under L11 (19.465″), followed by B11 (19.096″), B12 (16.406″), L23 (16.381″), and L13 (16.128″); For the lateral incisor, the maximum was observed under L33 (20.231″), followed by L23 (16.767″), L11 (11.835″), B33 (10.729″), and L13 (9.905″); For the canine, the maximum was observed under L11 (7.703″), followed by L33 (6.909″), L23 (5.636″), L13 (3.904″), and B13 (1.801″). With the same mini-implant and loading sites, linguoincisal loading resulted in greater changes in labiolingual angulation of the maxillary incisors compared to labial loading, such as L11 and B11, L13 and B13, L23 and B23, as well as L33 and B33.

### Effects of different intrusion modes on the mesiodistal angulation of canine

As displayed in Fig. 4 (c), the changes in mesiodistal angulation of canine were positive under labial loading, with the maximum observed under B13 (1.144″). While the changes were negative under linguoincisal loading except for condition L14, with the minimum observed under L11 (-3.319″).

### Effects of different intrusion modes on the displacement tendency of CA

**Fig. 5:**
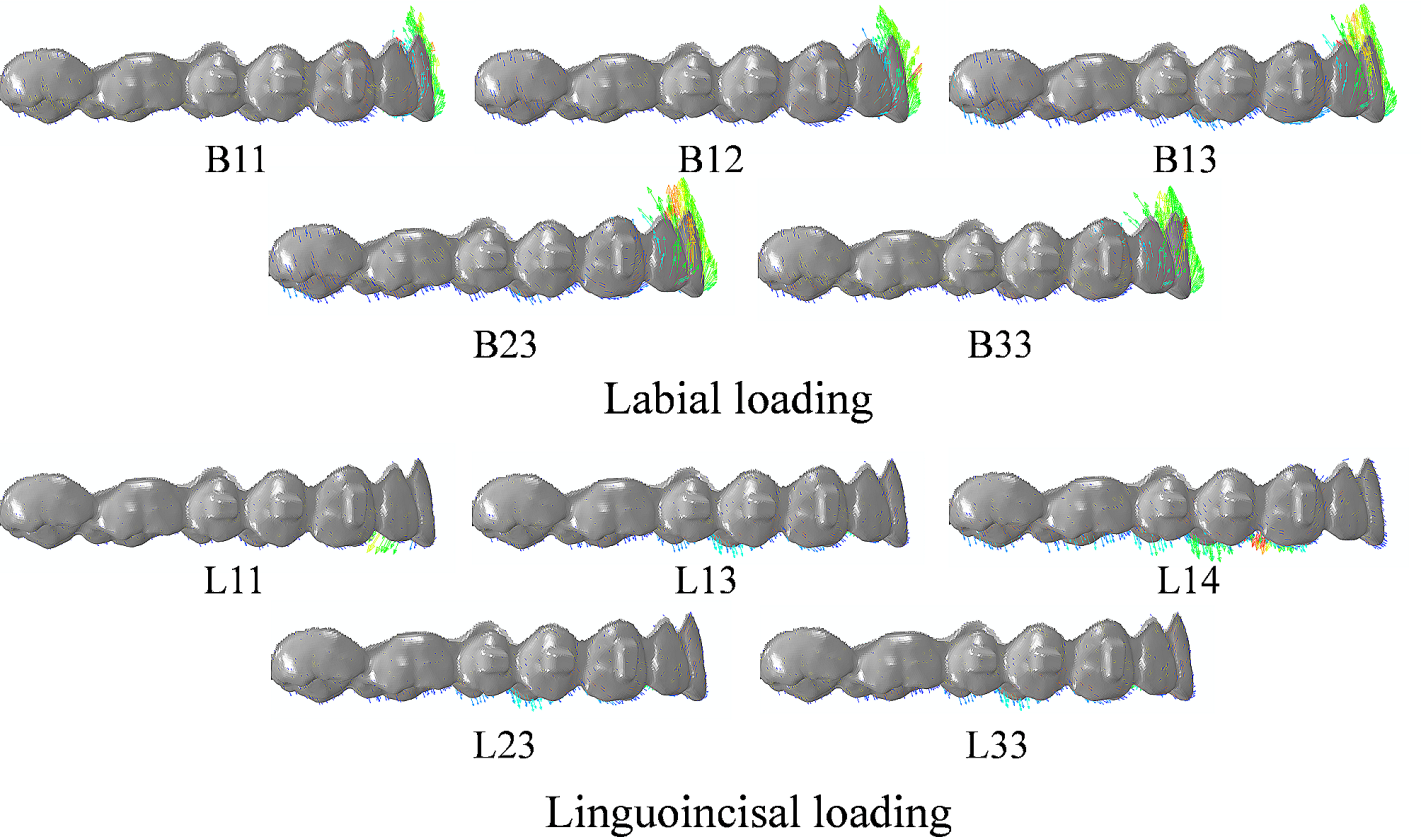
Displacement tendencies of CA under different conditions

As displayed in Fig. 5, the CA at the posterior teeth was displaced downward under all conditions. For the CA at the anterior teeth, its labial surface was displaced upward under all conditions, with a more obvious tendency under labial loading. Its lingual surface was displaced upward under labial loading but downward under linguoincisal loading.

### Effects of different intrusion modes on the maximum principal stress of PDLs

**Fig. 6:**
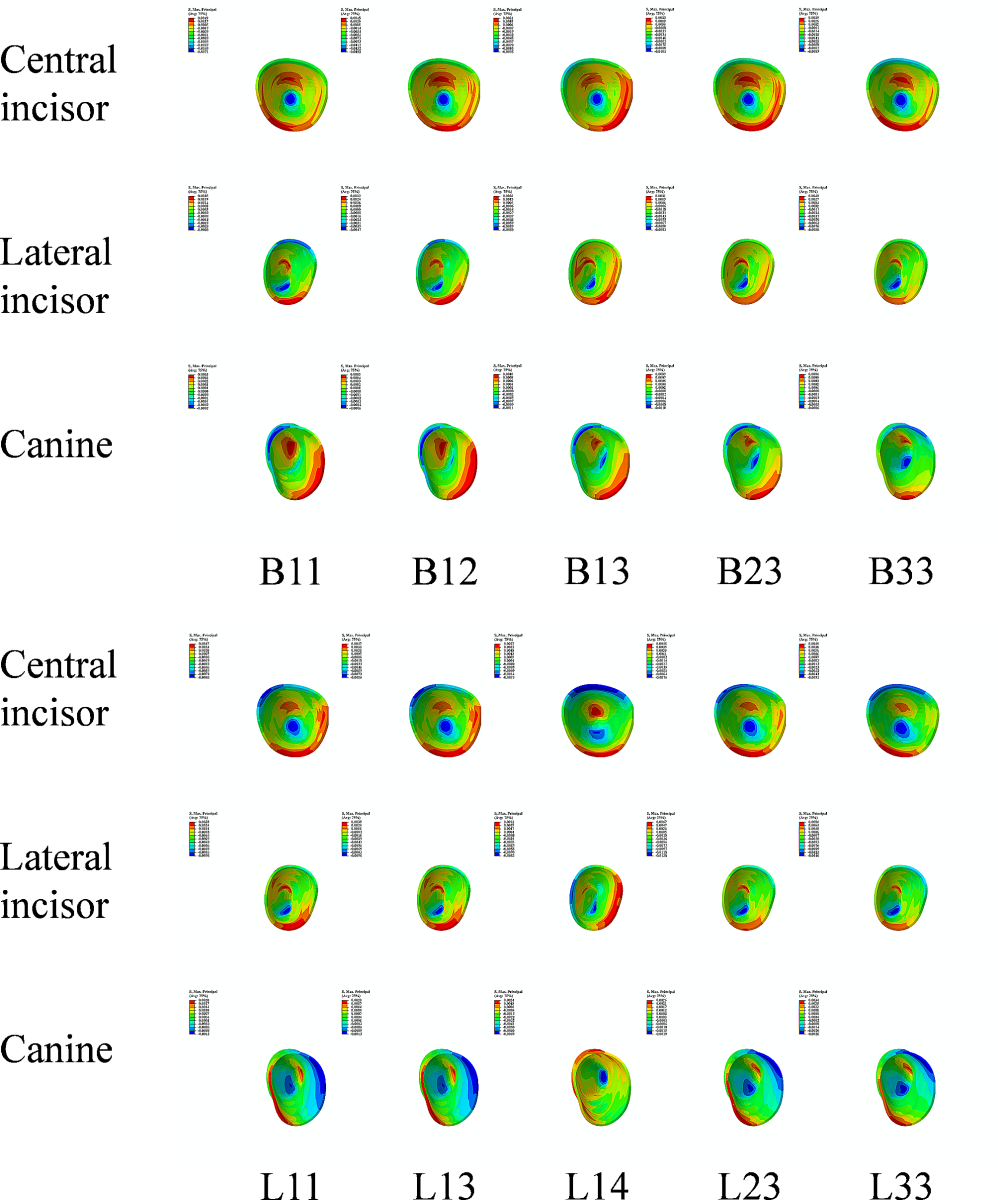
Maximum principal stress distribution of PDLs (MPa)

As displayed in Fig. 6, the stress was mainly concentrated in the cervical and apical regions. The peak values of PDL compressive stress were listed in Table 3. Among all conditions, the peak stress values of the central incisor were higher under B11 (17.1 KPa) and L11 (23.5 KPa).

**Table 3:** The peak values of PDL compressive stress of each tooth under different conditions (KPa)

Condition	Central incisor	Lateral incisor	Canine
B11	17.1	3.0	0.2
B12	15.2	4.7	0.6
B13	9.5	8.0	1.1
B23	10.2	9.2	1.0
B33	9.5	8.8	0.6
L11	23.5	3.5	3.6
L13	8.6	9.6	1.3
L14	1.9	8.3	6.9
L23	7.6	13.8	1.9
L33	5.2	14.6	2.6

## Discussion

The intrusion of maxillary anterior teeth has always posed challenges in orthodontics, while the application of mini-implants has significantly improved the intrusion efficiency [3, 11, 12]. Moreover, different mini-implant sites and loading sites can be selected to design different intrusion modes [6, 7, 11, 12]. Compared to fixed appliance, the CA offers a wider range of loading sites. For example, intrusive forces can be applied to the labial or lingual side of the CA by 3D-printed buttons or precision cuts [11, 12, 20], or applied directly to the maxillary anterior teeth by bonded buttons [12]. Different intrusion modes will cause different effects, which is significantly important for achieving precise and safe tooth movement in clinical practice. However, relevant studies are limited and there is a lack of reliable references in clinical practice.

Arturo et al. [6]. compared the effects of two intrusion modes in fixed orthodontic treatment in clinical practice and found that mini-implants between the central incisors were more advantageous for increasing the labiolingual angulation of the maxillary incisors, while mini-implants between the lateral incisor and canine were more advantageous for the correction of maxillary anterior teeth intrusion and overbite. Liu et al. [12]. simulated maxillary anterior teeth retraction in clear aligner treatment with and without anterior mini-implant using three-dimensional finite element analysis. The mini-implant was designed between maxillary central incisors and the loading sites were designed on the labial side of the maxillary incisors or the lingual side of the CA. This study found that anterior mini-implant can effectively achieve incisor intrusion and palatal root torquing, and the linguoincisal loading is more effective than the labial loading.

At present, the research methods in oral biomechanics include clinical studies [6, 11], in vitro studies [21] and FEA studies [12, 17]. Due to the complex structure of human tissue, there are many factors influencing the intrusion effects of maxillary anterior teeth, such as the labiolingual angulation [22] and crown-root ratio [22] of maxillary anterior teeth, alveolar bone height [17, 22], intrusive force value [6, 12] and patient compliance. Therefore, it is challenging to control variables in clinical studies. In vitro studies, while allowing for the control of variables, fail to simulate the periodontal tissue and measure the tooth displacement due to the rigid connection between the tooth model and the sensors [21]. FEA studies can accurately control variables and have been proved to be able to simulate real clinical situations, which is a commonly used method in biomechanical research [23]. Consequently, FEA was used in the study and a patient with normal labiolingual angulation and crown-root ratio of maxillary anterior teeth was selected as the subject. The intrusive force value suggested by Burstone [18] and Cho [17] was adopted and this study further investigated the effectiveness of different intrusion modes of maxillary anterior teeth with mini-implants in clear aligner treatment.

In FEA, the measurement methods of different scholars were not the same. To measure the intrusion amount, some scholars chose the incisal center, canine cusp and apical center as the measuring points [12, 17]. However, these measurements would be affected by the changes in labiolingual and mesiodistal angulation of the anterior teeth [3, 6, 18]. For example, an increase in labiolingual angulation would result in greater crown intrusion and less root intrusion. In this study, the vertical displacement of CR points was measured, which was less affected by these angulation changes [6, 18]. To measure the change in labiolingual angulation, Cho et al. [17]. established a global coordinate and measured the angle of teeth axes on the sagittal plane before and after loading. However, our study found that the method may be affected by the tooth position in the dental arch. As displayed in Fig. 3, the result measured in the global coordinate represented the rotation angle of each tooth around the x1-axis. While the actual change in labiolingual angulation for each tooth was the rotation angle around the line connecting mesiodistal contact points, namely the x2-axis. Due to the natural curvature of the dental arch, the distally positioned tooth exhibited a greater angle between the x1-axis and the x2-axis, resulting in a greater difference between the two measurement results. Therefore, this study established the local coordinates for each tooth, and measured the angle of teeth axes on sagittal planes separately. This method could eliminate the influence of tooth position in the dental arch, exhibiting better accuracy.

The two null hypotheses proposed and investigated in this study were all rejected. The first hypothesis stated that different intrusion modes do not affect the displacement tendency of the maxillary anterior teeth and CA. While the results showed that the intrusion amount and labiolingual angulation of the anterior teeth, the mesiodistal angulation of the canine, as well as the displacement of CA were all different under different intrusion modes. The second hypothesis stated that different intrusion modes do not affect the stress of the PDLs, which was also rejected as different intrusion modes lead to different distribution and peak values of PDL maximum principal stress.

As displayed in Fig. 4 (a), the intrusion amount of maxillary anterior teeth was found to be influenced by the mini-implant site, loading site, and loading mode. Among these factors, the loading site had the greatest influence. As the loading site gradually moved from between the central incisors to between the lateral incisor and canine, the intrusion amount of the central incisor gradually decreased and that of the canine gradually increased, aligning with the findings of Cho et al [17]. Additionally, the intrusion amount was affected by the mini-implant site. For example, the intrusion amount of the central incisor under condition B23 was greater than that under B13 and B33. This was because the mini-implant site, along with the loading site, determined the force lines. As the angle between the force lines and the tooth axes increased, there was a decrease in the intrusive component of the forces, leading to less intrusion amount. At the same time, the intrusion amount was also affected by the loading mode. Under labial loading, the maxillary anterior teeth consistently exhibited intrusion. Under linguoincisal loading, the canine exhibited extrusion when the loading sites were between the central incisors or between the central and lateral incisor (e.g., L11, L13, L23, and L33), and the central incisor exhibited extrusion when the loading sites were between the lateral incisor and canine (e.g., L14). The extrusion amount of the canine was greater than that of the central incisor. This phenomenon may be due to the downward displacement tendency of the CA under linguoincisal loading, resulting in the generation of extrusion forces. A greater retention between the CA and the tooth would result in higher extrusion forces. This study designed rectangular retention attachments on the labial surface of the canine, which may account for its greater extrusion amount. This phenomenon may also be related to the material properties of CA. In this study, the elastic modulus of CA was set 528 MPa [15, 20], which is a highly elastic material prone to deformation. Further studies are needed to determine whether the extrusion of the central incisor and canine would still occur when using a CA model with a larger elastic modulus. In clinical practice, in order to solve the problems of excessive exposure of the maxillary anterior teeth and gummy smile, significant intrusion of the central incisors and lateral incisors is necessary [1]. In such situations, the loading sites could be designed between the central and lateral incisor (e.g., the intrusion modes under B13, B23, B33, L13, and L23). Under these conditions, the intrusion amount of the central incisor and lateral incisor was greater than 0.5 μm, while the intrusion amount of the canine was less than 0.05 μm.

**Fig. 7:**
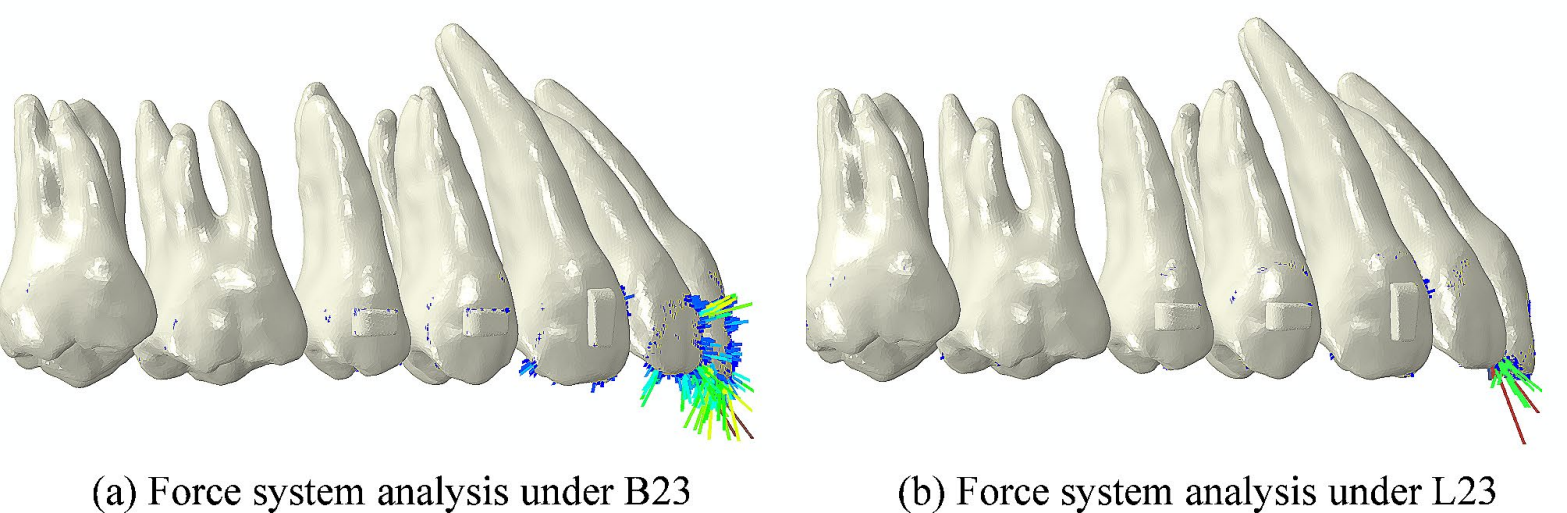
Force system analysis under B23 and L23. The arrows indicate the concentration points of forces on the dentition. The color and length of the arrows represent the force value. The darker the warm color and the longer the length, the greater the force value. (**a**) Force system analysis under B23, in addition to the intrusive forces at the incisal edges, the labial surfaces of incisors were also subjected to compressive forces. (**b**) Force system analysis under L23, the incisors were mainly subjected to intrusive forces at the incisal edges

In addition to the effects of different intrusion modes on the intrusion amount, it is also necessary to consider their effects on the labiolingual angulation of the maxillary anterior teeth. In fixed orthodontic treatment, intrusive forces are applied on the brackets or archwire. So the force lines are mostly located on the labial side of the CRs of teeth, thereby resulting in increased labiolingual angulation [6, 24]. However, in clear aligner treatment, the aligner wraps around the outer surface of the teeth, making it difficult to predict the aligner-teeth contact positions after applying intrusive forces, which increases its biomechanical complexity [13]. As displayed in Fig. 4 (b), this study found that the labiolingual angulation of the maxillary anterior teeth increased under all the intrusion modes. Compared to labial loading, the labiolingual angulation of incisors increased more under linguoincisal loading. In order to explore the underlying reasons, the force systems on maxillary incisors under different conditions were further analyzed. Taking conditions B23 and L23 as examples (as displayed in Fig. 7), under linguoincisal loading, the incisors were mainly subjected to the intrusive forces at the incisal edges. While under labial loading, in addition to the intrusive forces, the labial surfaces of incisors were also subjected to compressive forces. This difference may be due to the different displacement tendencies of CA. Under linguoincisal loading, elastic bands were worn from the mini-implants to the lingual side of CA and went beneath the incisal edges. The lingual side of CA was displaced downward and the labial side was displaced upward. This resulted in intrusive forces at the incisal edges, generating labial crown torque. Under labial loading, elastic bands were worn from the mini-implants to the labial side of CA directly. Since the mini-implants were positioned lingually to the crown of incisors in sagittal direction, the CA was displaced upward and backward. The backward displacement produced compressive forces on the labial surfaces of incisors, generating lingual crown torque, which mitigated the increase in labiolingual angulation. These were just preliminary findings and further clinical validation is needed. Additionally, this study found that the labiolingual angulation of the central incisor increased the most when both the mini-implant and loading sites were located between the central incisors (i.e., the intrusion modes under B11 and L11), which was advantageous for the torque control in extraction cases. And the labiolingual angulation of the maxillary anterior teeth exhibited minimal increase when the mini-implant site was located between the central incisors and the loading sites were located between the lateral incisor and canine (i.e., the intrusion mode under L14), which was beneficial for achieving true intrusion of the maxillary anterior teeth.

The mesiodistal angulation of the canine is crucial for occlusal function. Some scholars found that in clear aligner treatment, the mesiodistal angulation control of the canines was important, ranking only second to the labiolingual angulation control of the central incisors [15]. Distal crown tipping of the canine would lead to the roller-coaster effect and make the resolution of anterior deep overbite more difficult [15]. Therefore, the change in mesiodistal angulation of the canine was analyzed in this study. As displayed in Fig. 4 (c), the canine crown tended to tip mesially under labial loading, while distally under linguoincisal loading. Consequently, in clinical practice, if linguoincisal loading is chosen to intrude the maxillary anterior teeth, it is advisable to pre-design greater mesial crown tipping or distal root tipping of the canine. And the mesiodistal angulation of the canine should be monitored regularly to prevent the roller-coaster effect.

Accurate tooth movement is crucial in orthodontic treatment, and the safety is also of great importance. Root resorption (RR) is a common complication and the intrusion movement is more likely to cause RR due to the stress concentration at the root apex [25]. In FEA, the risk of RR can be assessed by evaluating the maximum principal stress value and distribution of the PDL [26]. However, there is no consensus on the optimal stress value of the PDL during tooth movement. Initially, Schwarz et al. [27]. (1932) suggested that the optimal stress value was 20–26 g/cm² (1.96–2.55 KPa), which was just able to overcome the pressure of the PDL capillaries. In 2007, Hohmann et al. [28]. believed that RR would occur only when the PDL stress exceeded 4.7 KPa. Another study proposed that the PDL stress should not exceed the systolic heart pressure, which was approximately 16 KPa [29]. In this study, the peak values of the PDL compressive stress under different intrusion modes were compared with the reported maximum risk value (16 KPa) to evaluate the risk of RR. This study found that the stress was primarily concentrated in the cervical and apical regions of the PDLs under all conditions. The peak values of the central incisor under B11 and L11 were greater than 16 KPa. This suggests that the intrusive force value should be properly reduced to prevent RR when positioning the mini-implant and loading sites between the central incisors. Although relatively lower peak values were observed under other conditions, X-ray should still be taken regularly to monitor the occurrence of RR. In addition, for the central incisor under L14 and the canine under B11, B12, B13, B23, B33, L13 and L23, the peak values of PDL compressive stress were lower than the minimum capillary pressure (1.96 KPa), indicating that movement of these teeth may not actually occur [27].

In clear aligner treatment, when the anterior teeth are intruded with the aligner, the posterior teeth will be extruded as anchorages [14, 30]. In order to improve the intrusion efficiency and prevent the CA from off-tracking, it is necessary to design retention attachments on the posterior teeth [14, 30]. This study found that, when mini-implant anchorage was used, there still existed off-tracking tendencies of the CA at the posterior teeth, which should be paid attention to in clinical practice and more retention attachments should be designed if necessary. In addition, when designing linguoincisal loading, it is important to consider the off-tracking tendency of the lingual side of CA. This can result in reduced wrapping of CA, thereby affecting the efficiency of designed tooth movement.

However, there are several limitations in this study. Firstly, the study only analyzed the effect on the anterior teeth, while the effect on the posterior teeth remained to be evaluated. Secondly, the FEA results can only show the initial effect and cannot reflect the long-term periodontal remodeling. Finally, the intraoral environment, masticatory forces, and deformation of CA in the process of wearing and removing may affect the results. So, further clinical studies are needed for validation.

## Conclusions

Under the experimental conditions of this study, the following conclusions can be drawn:

1. The intrusion amount, labiolingual and mesiodistal angulations of the maxillary anterior teeth were affected by the mini-implant site, loading site and loading mode.
2. All the maxillary anterior teeth would be intruded under labial loading, while extrusion of the central incisor and canine occurred under linguoincisal loading.
3. The labiolingual angulation of maxillary incisors would increase under all the intrusion modes, with greater increases under linguoincisal loading.
4. The canine crown would tip mesially under labial loading, while distally under linguoincisal loading.

## Data Availability

The authors confirm that the data supporting the findings of this study are available within the article.

## Abbreviations

PDL: Periodontal ligament
CA: Clear aligner
FEA: Finite element analysis
CBCT: Cone beam computed tomography
CR: Center of resistance
RR: Root resorption

## References

[1] Saga AY, Araújo EA, Antelo OM (2020). Nonsurgical treatment of skeletal maxillary protrusion with gummy smile using headgear for growth control, mini-implants as anchorage for maxillary incisor intrusion, and premolar extractions for incisor retraction[J]. Am J Orthod Dentofac Orthop.

[2] Zengiski ACS, Basso IB, Cavalcante-Leão BL (2022). Effect and longevity of botulinum toxin in the treatment of gummy smile: a meta-analysis and meta-regression[J]. Clin Oral Invest.

[3] Bardideh E, Tamizi G, Shafaee H (2023). The effects of intrusion of anterior teeth by Skeletal Anchorage in Deep Bite patients; a systematic review and Meta-Analysis[J]. Biomimetics.

[4] Yun S, Park JH, Chang NY (2021). Craniomaxillofacial changes using high-pull J-Hook headgear and Mini-implant Anchorage in adolescents: a structural superimposition Method[J]. J Clin Pediatr Dentistry.

[5] Alharbi F, Almuzian M, Bearn D (2018). Miniscrews failure rate in orthodontics: systematic review and meta-analysis[J]. Eur J Orthod.

[6] Arturo, Gutiérrez-Zubeldia L, López-García R (2020). One versus two anterior miniscrews for correcting upper incisor overbite and angulation: a retrospective comparative study[J]. Prog Orthodont.

[7] El Namrawy MM, El Sharaby F, Bushnak M (2019). Intrusive Arch versus Miniscrew-supported intrusion for deep bite Correction[J]. Open Access Macedonian J Med Sci.

[8] Lyu X, Cao X, Chen L (2023). Accumulated biomechanical effects of mandibular molar mesialization using clear aligners with auxiliary devices: an iterative finite element analysis[J]. Prog Orthodont.

[9] Castroflorio T, Sedran A, Parrini S (2023). Predictability of orthodontic tooth movement with aligners: effect of treatment design[J]. Prog Orthodont.

[10] Al-balaa M, Li H, Mohamed MA (2021). Predicted and actual outcome of anterior intrusion with Invisalign assessed with cone-beam computed tomography[J]. Am J Orthod Dentofac Orthop.

[11] Yan X, Zhang X, Ren L (2023). Effectiveness of clear aligners in achieving proclination and intrusion of incisors among class II division 2 patients: a multivariate analysis[J]. Prog Orthodont.

[12] Liu L, Zhan Q, Zhou J (2021). Effectiveness of an anterior mini-screw in achieving incisor intrusion and palatal root torque for anterior retraction with clear aligners[J]. Angle Orthod.

[13] Elshazly TM, Bourauel C, Aldesoki M (2022). Computer-aided finite element model for biomechanical analysis of orthodontic aligners[J]. Clin Oral Invest.

[14] Li Y, Xiao S, Jin Y (2023). Stress and movement trend of lower incisors with different IMPA intruded by clear aligner: a three-dimensional finite element analysis[J]. Prog Orthodont.

[15] yin Zhu G, Zhang B, Yao K (2023). Finite element analysis of the biomechanical effect of clear aligners in extraction space closure under different anchorage controls[J]. Am J Orthod Dentofac Orthop.

[16] Burstone CR (1977). Deep overbite correction by intrusion[J]. Am J Orthod Dentofac Orthop.

[17] Wang Q, Dai D, Wang J (2022). Biomechanical analysis of effective mandibular en-masse retraction using class II elastics with a clear aligner: a finite element study[J]. Prog Orthodont.

[18] Ammar HH, Ngan P, Crout RJ (2011). Three-dimensional modeling and finite element analysis in treatment planning for orthodontic tooth movement[J]. Am J Orthod Dentofac Orthop.

[19] Cho SM, Choi SH, Sung SJ (2016). The effects of alveolar bone loss and miniscrew position on initial tooth displacement during intrusion of the maxillary anterior teeth: Finite element analysis[J]. Korean J Orthod.

[20] Burstone CJ, Pryputniewicz RJ (1980). Holographic determination of centers of rotation produced by orthodontic forces[J]. Am J Orthod Dentofac Orthop.

[21] Mao B, Tian Y, Xiao Y (2023). The effect of maxillary molar distalization with clear aligner: a 4D finite-element study with staging simulation[J]. Prog Orthodont.

[22] Spanier C, Schwahn C, Krey KF (2023). Fused filament fabrication (FFF): influence of layer height on forces and moments delivered by aligners—an in vitro study[J]. Clin Oral Invest.

[23] Geramy A, Sodagar A, Hassanpour M. Three-Dimensional analysis using finite element method of anterior teeth inclination and center of resistance location[J]. Chin J Dent Res. 2014;17(1):37–42. 25028688

[24] Kale Varlık S, Onur Alpakan Ö, Türköz Ç (2013). Deepbite correction with incisor intrusion in adults: a long-term cephalometric study[J]. Am J Orthod Dentofac Orthop.

[25] Weltman B, Vig KWL, Fields HW (2010). Root resorption associated with orthodontic tooth movement: a systematic review[J]. Am J Orthod Dentofac Orthop.

[26] Cheng Y, Liu X, Chen X (2022). The three-dimensional displacement tendency of teeth depending on incisor torque compensation with clear aligners of different thicknesses in cases of extraction: a finite element study[J]. BMC Oral Health.

[27] Schwarz AM (1932). Tissue changes incident to tooth movement[J]. Int J Orthod.

[28] Hohmann A, Wolfram U, Geiger M (2007). Periodontal Ligament Hydrostatic pressure with areas of Root Resorption after application of a continuous Torque Moment[J]. Angle Orthod.

[29] Rygh P (1973). Ultrastructural changes in pressure zones of human periodontium incident to orthodontic tooth movement[J]. Acta Odontol Scand.

[30] Liu L, Song Q, Zhou J (2022). The effects of aligner overtreatment on torque control and intrusion of incisors for anterior retraction with clear aligners: a finite-element study[J]. Am J Orthod Dentofac Orthop.

